# Psychometric network analysis of the Patient Health Questionnaire-4 (PHQ-4) in Paraguayan general population

**DOI:** 10.1186/s41155-024-00299-x

**Published:** 2024-04-15

**Authors:** Tomás Caycho-Rodríguez, Aaron Travezaño-Cabrera, Julio Torales, Iván Barrios, Lindsey W. Vilca, Antonio Samaniego-Pinho, Rodrigo Moreta-Herrera, Mario Reyes-Bossio, Nicol A. Barria-Asenjo, Jesús Ayala-Colqui, Cirilo H. Garcia-Cadena

**Affiliations:** 1 Facultad de Psicología, Universidad Científica del Sur, Campus Villa II, Ctra. Panamericana S 19, Villa EL Salvador, Lima, Peru; 2 Escuela de Psicología, Universidad Peruana Unión, Lima, Perú; 3 Cátedra de Psicología Médica, Facultad de Ciencias Médicas, Universidad Nacional de Asunción, San Lorenzo, Paraguay; 4 Instituto Regional de Investigación en Salud, Universidad Nacional de Caaguazú, Coronel Oviedo, Paraguay; 5 Facultad de Ciencias Médicas, Universidad Sudamericana, Pedro Juan Caballero, Paraguay; 6 Cátedra de Bioestadística, Facultad de Ciencias Médicas, Universidad Nacional de Asunción, Santa Rosa del Aguaray Campus, Santa Rosa del Aguaray, Paraguay; 7 South American Center for Education and Research in Public Health, Universidad Norbert Wiener, Lima, Peru; 8 Carrera de Psicología, Facultad de Filosofía, Universidad Nacional de Asunción, Asunción, Paraguay; 9 Escuela de Psicología, Pontificia Universidad Católica del Ecuador, Ambato, Ecuador; 10 Facultad de Psicología, Universidad Peruana de Ciencias Aplicadas, Lima, Peru; 11 Departamento de Ciencias Sociales, Universidad de Los Lagos, Osorno, Chile; 12 Universidad Tecnólogica del Perú, Lima, Peru; 13 Facultad de Psicología, Universidad Autónoma de Nuevo León, Monterrey, Mexico

**Keywords:** Network psychometric analysis, Reliability, Invariance, Patient Health Questionnaire-4, Validity

## Abstract

**Background:**

Depression and anxiety are two of the most prevalent and disabling mental disorders worldwide, both in the general population and in outpatient clinical settings.

**Objective:**

This study aimed to analyze the psychometric properties of the Patient Health Questionnaire-4 (PHQ-4) based on network analysis metrics.

**Methods:**

A total of 911 Paraguayans (23.71% women and 76.29% men; mean age 31.25 years, SD = 10.63), selected by non-probabilistic convenience sampling, participated in the study. Network analysis was used to evaluate the internal structure, reliability, and measurement invariance between men and women.

**Results:**

The results revealed that the PHQ-4 is a unidimensional measure through Exploratory Graph Analysis (EGA). Reliability, through structural consistency, identified that 100% of the time, only a single dimension was obtained, and all items remained stable, as they were always replicated within the empirical dimension. The unidimensional structure has shown evidence of configural invariance; therefore, the network structure functioned equally among the different sex groups.

**Conclusion:**

The PHQ-4 presented optimal preliminary evidence of validity based on its internal structure, reliability, and invariance between sexes. Therefore, it may be useful as an accurate and brief measure of anxiety and depressive symptoms in the Paraguayan context.

## Introduction

Depression and anxiety are two of the most prevalent and disabling mental disorders worldwide (Christodoulaki et al., 2022; Wicke et al., 2022), both in the general population and in outpatient clinical settings (Ghaheri et al., 2020). According to the Global Burden of Disease Study, depression and anxiety rank fifth and ninth among the leading causes of years lost owing to disability, respectively (Wicke et al., 2022). Prior to the COVID-19 pandemic, approximately 260 million people worldwide were reported to have anxiety and more than 300 million were reported to have depression (World Health Organization, 2017). During the pandemic, the prevalence of depression (28.18% [95% CI:23.81–32.54]) and anxiety (29.57% [95% CI:24.67–34.47]) was high compared with normal pre-pandemic times (Mahmud et al., 2023). In Latin America, during the pandemic, the prevalence of anxiety was 35% as was the prevalence of depression (35%), with a higher prevalence of mental health symptoms in South America (36%) than in Central America (28%). In Paraguay, different studies have reported that 34.4% (Torales et al., 2022a, 2022b) and 32% (Caycho-Rodríguez et al., 2021) of the samples derived from the general population suffered from severe symptoms of depression and generalized anxiety disorders, respectively. The High Frequency Surveys (HFS) conducted by the World Bank in 2021 indicated that Paraguay ranked fourth (tied with Ecuador and Bolivia) among the countries with the most worrisome results in the Mental Health Vulnerability Index during the pandemic (Canavire-Bacarreza & Recalde-Ramírez, 2022).

It has been estimated that only approximately 2.2% of people with depressive and anxious symptoms seek medical attention (Ohayon & Hong, 2006). Anxiety and depressive disorders are usually first diagnosed and treated in a primary care setting (Serrano-Blanco et al., 2010). However, in primary care, the time available for consultation is short, and health professionals only have approximately 5–10 min to diagnose, treat, and/or refer patients for further treatment (Cano-Vindel et al., 2018). For example, in the primary care setting, about 90% of physicians indicated the need for more time to make a diagnosis of depression compared to other illnesses (Wittchen & Pittrow, 2002). In addition, the relatively high prevalence of anxiety and depression in primary care tends to overload the daily practice of health professionals, which generates low rates of diagnosis and treatment (Cano-Vindel et al., 2018). This is expressed in the fact that health professionals identify only 39% of patients with depression (Thompson et al., 2000). Furthermore, other studies have indicated that less than 50% of episodes of depression and anxiety are correctly diagnosed (Mitchell et al., 2009; Parmentier et al., 2013). An incorrect or missed diagnosis is a serious problem as it decreases the likelihood of patients receiving appropriate treatments for anxiety and depression.

Currently, there are different instruments to measure the symptoms of depression and anxiety, such as the 17-item Hamilton Depression Rating Scale (HDRS; Hamilton, 1960), 14-item Hamilton Anxiety Rating Scale (HARS; Hamilton, 1959), Self-Rating Depression Scale (Zung, 1965), and Self-Rating Anxiety Scale (Zung, 1971), each consisting of 20 items. Recently, the Depression Anxiety and Stress Scale (DASS; Lovibond & Lovibond, 1995), 9-item Patient Health Questionnaire-9 (PHQ-9; Kroenke et al., 2001), and 7-item Generalized Anxiety Disorder Scale-7 (GAD-7; Spitzer et al., 2006) have also been developed. However, it has been suggested that to make mass screening more efficient or in highly crowded outpatient clinical settings, it is necessary to have shorter versions of the scales for application in the general and clinical populations (Caro-Fuentes & Sanabria-Mazo, 2023; Materu et al., 2020).

To avoid overburdening health professionals in primary care, the use of brief questions for screening for depressive and anxiety disorders is recommended (Wicke et al., 2022). Brief tools can help improve clinical outcomes by reducing misdiagnosis rates in primary care (Arroll et al., 2010; Schumann et al., 2012). Additionally, the use of brief measures allows for the detection of mental health disorders that support the implementation of early interventions (Mulvaney-Day et al., 2018). Thus, the presence of ultra-brief screening measures would help improve the resources of the primary healthcare system (Caro-Fuentes & Sanabria-Mazo, 2023). The Patient Health Questionnaire-4 (PHQ-4; Kroenke et al., 2009) is a brief screening measure of anxiety and depression used in the primary care setting. In addition, the PHQ-4 is useful in cohort and panel studies (Hajek & König, 2020). The PHQ-4 was designed based on the assumption that symptoms of depression and anxiety frequently coexist and aims to identify individuals who are experiencing one or both of these common symptoms (Kroenke et al., 2009).

The PHQ-4 consists of four items, two measuring depressive symptoms derived from the Patient Health Questionnaire-9 (PHQ-9; Kroenke & Spitzer, 2002) and two measuring anxiety symptoms from the General Anxiety Disorder-7 (GAD-7; Spitzer et al., 2006). The PHQ-4 has demonstrated high sensitivity and specificity in detecting depression and anxiety (Gilbody et al., 2008; Kroenke et al., 2009). Unlike other screening measures, the PHQ-4 has several advantages. As the PHQ-4 is a self-report measure, it allows the direct assessment of depression and anxiety levels from people's own perspective (Hartung et al., 2017). In addition, as mentioned above, the PHQ-4 is a brief measure whose items are easily worded and can be answered in a short time (Mitchell, 2010). Other measures with a greater number of items generate challenges, especially in those with cognitive functioning problems (Renovanz et al., 2019). However, it has been indicated that brief two- or four-item measures, such as the PHQ-4, outperform single-item measures (Mitchell & Coyne, 2007). Finally, the PHQ-4 is not a definitive diagnostic measure; however, the results derived from its application motivate further studies on mental health problems (Kroenke et al., 2009).

The PHQ-4 has been translated into different languages including German (Löwe et al., 2010; Wicke et al., 2022), Greek (Christodoulaki et al., 2022), Korean (Kim et al., 2021), Swahili (Materu et al., 2020), Arabic (Kliem et al., 2016), Persian (Ahmadi et al., 2019), Austrian, Croatian, Lithuanian, Portuguese, Swedish (Kazlauskas et al., 2023), and Spanish (Kocalevent et al., 2014; López Guerra et al., 2022). Regarding the psychometric evidence of the PHQ-4 since its original development (Kroenke et al., 2009), the presence of two factors (anxiety and depression) and adequate reliability have been suggested. The two-factor structure, adequate reliability, and evidence of measurement invariance across different ages, genders, and other groups have been replicated in different countries and population groups (Christodoulaki et al., 2022; Kazlauskas et al., 2023; Khubchandani et al., 2016; Kim et al., 2021; Kocalevent et al., 2014; Lenz & Li, 2022; Lopez Guerra et al.,^ 2022^; Löwe et al., 2010; Mendoza et al., 2022; Mills et al., 2015).

Despite the consistent presence of these two factors in the PHQ-4, it has been suggested that this structure may not be completely adequate (Kim et al., 2021). This has also been observed in a study conducted in Tanzania (Materu et al., 2020), where the results of confirmatory factor analysis (CFA) indicated that all items of the PHQ-4 significantly clustered into a single factor. However, the same study also suggested the presence of two factors (anxiety and depression) from an exploratory approach, using principal component analysis with varimax rotation. These two procedures are part of the package known as Little Jiffy (Dominguez-Lara & Merino-Soto, 2016), which has been reported in psychometric literature because of its large intrinsic limitations (Ferrando & Anguiano-Carrasco, 2010; Lloret-Segura et al., 2014).

The reliability estimate of the two-dimensional model, using Cronbach's alpha coefficient, ranged from 0.75 to 0.87, and with the omega coefficient ranged from 0.83 to 0.92 in different cultural contexts (Christodoulaki et al., 2022; Kazlauskas et al., 2023; Khubchandani et al., 2016; Kim et al., 2021; Kocalevent et al., 2014; Lopez Guerra et al., 2022; Löwe et al., 2010; Mendoza et al., 2022; Mills et al., 2015). Similarly, the unidimensional model of the PHQ-4 presented values of Cronbach's alpha and Omega coefficient that varied between 0.82 and 0.91 among different sexes, ages, ethnicities, and other groups (Lenz & Li, 2022).

Regarding the measurement invariance of the PHQ-4, previous studies have shown that the fit indices of the two-dimensional model are consistent despite greater model constraints. Specifically, all previous studies demonstrated that the configural (unrestricted) model presented good fit indices, and that the metric (with restriction of factor loadings between groups) and scalar (with restriction of item intersections between groups) invariance models when comparing male and female groups presented ΔCFI lower than 0.01 and a ΔRMSEA lower than 0.15 (Cano-Vindel et al., 2018; Christodoulaki et al., 2022; Kazlauskas et al., 2023; Kocalevent et al., 2014; Mendoza et al., 2022). Likewise, a multigroup analysis of the unidimensional model of the PHQ-4 indicated that scalar invariance between male and female groups can also be assumed (Lenz & Li, 2022).

### Psychometric network analysis

So far, what is known about the factor structure, reliability, and invariance of the PHQ-4 is based on latent variable models derived from classical test theory (CTT). However, in latent-variable models, the decision regarding factors obtained through exploratory factor analysis tends to be subjective. In this sense, determining the latent factor structure can lead to a lack of consensus in defining and interpreting the obtained factors (Bollen, 2002; Borsboom et al., 2003). Operationally defining latent factors tends to be a subjective process because it is the researcher who determines the latent factors (Bock, Goode, & Webb, 2003). In addition, there is no certainty that latent variables are directly related to psychological attributes. Data-based latent variables may be influenced by the sample and may not fully represent a psychological attribute (Bollen, 2002). Although this practice is common in self-report measures such as the PHQ-4, it is necessary to complement the findings with evidence provided by alternative and contemporary methods such as Item Response Theory (IRT) and Network Analysis (NA) (Dias et al., 2023). Both psychometric network models and latent variable models are alternatives, as they can be applied to describe or explain the variance–covariance structures of different variables of interest (McFarland, 2020).

NA is an analytical tool that provides another way to conceptualize and evaluate different aspects related to health (Luke & Harris, 2007). For NA, psychopathological disorders can be considered as a complex, dynamic, and interchangeable system consisting of symptoms or behaviors that interact with each other and are not only causes or effects of a disorder (Borsboom, 2008; Borsboom & Cramer, 2013; McNally, 2016). In psychology, NA allows the identification of observable psychological nodes or variables, such as symptoms, behaviors, or cognitions, among others, and edges, which are the statistical relationships between the nodes (Epskamp et al., 2018; Hevey, 2018; McNally, 2016). NA allows for the identification of the most central symptoms or nodes that can be useful for diagnosis and treatment planning (Fonseca-Pedrero, 2018). Also, "bridging" symptoms that relate two network structures can be observed, which is valuable when considering comorbidity between psychological disorders (Costantini & Perugini, 2017). One of the advantages of NA is the presence of a diagram that allows visualization of the relationships between nodes and edges (partial correlations), where the thickness of the latter indicates the strength of the relationship (Epskamp & Fried, 2016).

NA postulates can be applied to psychometrics (Epskamp et al., 2018). In the same way as latent variable models, psychometric network analysis allows for an exploratory estimation of the underlying interconnectedness of observed data. However, unlike the traditional latent-variable model, psychometric network analysis does not assume the presence of latent factors or constraints derived from the principle of local independence (Schmank et al., 2019). The psychometric network model suggests that symptoms or traits are not due to a common latent cause, but arise from bidirectional relationships among themselves (Cramer et al., 2012). From this perspective, latent variables are not required to explain the presence of covariation among questionnaire items (Borsboom et al., 2009). In this sense, the relationship between a questionnaire and the latent variable is mereological, where the questionnaire items do not measure the latent variable but are part of it (Borsboom, 2008). Therefore, the latent variable exists as a stable network of dynamic components that activate each other (Schmittmann et al., 2013).

In the psychometric network model, nodes represent the items of a questionnaire and edges represent the relationships between items (Epskamp & Fried, 2018; Epskamp et al., 2016). Using exploratory graph analysis (EGA; Golino & Epskamp, 2017), latent dimensions can be identified in network models, based on a clustering algorithm for weighted networks (Pons & Latapy, 2006). In this manner, nodes are grouped into ordered and related subnetworks, where clusters are similar to latent variables (Epskamp et al., 2017). The psychometric approach allows the modeling of relationships between variables and complements the traditional latent variable approach (Ferguson & Alzheimer's Disease Neuroimaging Initiative, 2021). A psychometric network analysis provides a new interpretation of the emergence of dimensions (Soares et al., 2021). Psychometric networks report the degree to which items represent a dimension, demonstrating whether the components significantly measure a construct (Christensen et al., 2020). Likewise, structural consistency analysis provides additional information on traditional measures of internal consistency by combining internal consistency and homogeneity (Christensen et al., 2020). Finally, the graphical nature of psychometric networks can be intuitively interpreted by professionals without psychometric training (Soares et al., 2021).

The network approach is useful for determining the dimensionality of the PHQ-4, since it does not test alternative hypotheses of multidimensionality versus unidimensionality of the construct; rather, the data are freely expressed (Giuntoli & Vidotto, 2021). Moreover, compared to other methods of extracting the number of factors, EGA had 100% accuracy, whereas exploratory factor analysis had a mean accuracy of 10% to 49%, and confirmatory factor analysis had a mean accuracy of 74% based on Akaike's information criterion (Golino & Demetriou, 2017; Keith et al., 2016). The PHQ-4 and its components have mostly been tested in North American, European, and Asian populations. Therefore, there is little research on the PHQ-4 as an instrument for the mass screening of depressive and anxiety symptoms in the Latin American context. Thus, this study aimed to evaluate the psychometric properties of the PHQ-4 based on NA metrics, which is a growing field of research. Specifically, the evidence of validity based on internal structure, reliability, and measurement invariance was examined.

## Methods

### Participants

A total of 911 Paraguayans participated in this study, selected by non-probabilistic convenience sampling, based on the following inclusion criteria: a) being over 18 years of age, b) being of Paraguayan nationality, and c) accepting informed consent. The number of participants was determined using the iterative Monte Carlo method for NA. An a priori power of 0.80, a density of 0.40, four nodes, and a sensitivity of 0.60 were established (Constantin et al., 2023). These parameters suggested a total of 300 participants. This study significantly exceeded the recommended minimum number.

Of the participants, 23.71% were women and 76.29% were men. Their ages ranged from 18 to 60 years, with an average of 31.25 years (SD = 10.63). Most participants were single (66.0%), had completed university studies (53.4%), and had a steady job (55.3%). In addition, most participants lived in the city (88.3%) and reported no chronic diseases (84.2%). Table 1 shows the sociodemographic characteristics of the participants.

**Table 1:** Sociodemographic data of participants (*N* = 911)

Characteristic	*n*	%
Age (M ± SD)	24.3	7.9
Sex		
Female	216	23.7
Male	695	76.3
Social status		
Single	601	66.0
Married	207	22.7
Divorced	26	2.9
Partnered	71	7.8
Widowed	6	0.7
Educational level		
Primary, complete	4	0.4
Primary, incomplete	0	0.0
Secondary, complete	80	8.8
Secondary, incomplete	16	1.8
Technicature, complete	20	2.2
Technicature, incomplete	1	0.1
University, complete	486	53.4
University, incomplete	304	33.4
Employment		
Permanent job	504	55.3
Temporary job	155	17.0
Unemployed	252	27.7
Area of residence		
Urban	804	88.3
Rural	107	11.8
Chronic Diseases		
Yes	144	15.8
No	767	84.2

### Measures

#### Sociodemographic form

A specific survey was designed to obtain information on age, sex, marital status, educational level, work, residence, and presence of chronic diseases.

#### Patient Health Questionnaire-4

(PHQ-4; Kroenke et al., 2009). The PHQ-4 is a brief measure of anxiety and depressive symptoms and consists of four items. The first two items measure depressive symptoms ("During the past 2 weeks, how often have you been bothered by feeling discouraged, depressed, or hopeless?" and "During the past 2 weeks, how often have you been bothered by: feeling little interest or pleasure in doing things? ") and the next two items measure symptoms of anxiety ("During the past 2 weeks, how often have you been bothered by feeling nervous, anxious, or jittery?" and "During the past 2 weeks, how often have you been bothered by, not being able to stop worrying or not being able to control worry?" PHQ-4 corresponds to the first two items of the PHQ-9 (Kroenke et al., 2001) and GAD-7 (Spitzer et al., 2006). Both the PHQ-9 and GAD-7, and therefore their first two items, have already been used in a previous multinational study demonstrating adequate reliability in the Paraguayan sample (αPHQ-9 = 0.89 and αGAD-7 = 0.86), and the four items that make up the PHQ-4, two corresponding to the PHQ-9 and the other two from the GAD-7, presented high factor loadings (Caycho-Rodríguez et al., 2021). The Spanish version of the PHQ-4 used in the present study has been used in another study with a sample from another Latin American country (Ventura-León et al., 2023). Each of the four items has four response options: none = 0 to almost every day = 3. The total PHQ-4 score is obtained from the sum of the scores of each item. The score ranges from 0 to 12, with higher scores indicating a higher frequency of anxiety and depression symptoms.

### Procedure

Data were collected using Internet-mediated research procedures. An online questionnaire was constructed using the Google Form platform, which consisted of an informed consent form, a sociodemographic form and PHQ-4. First, the participants provided informed consent where the objective of the study, privacy and confidentiality of the data collected, and treatment of the data were indicated. All the participants were free to stop responding to the survey at any time. The online questionnaire was shared through different social networks (Facebook and Instagram) and emails. The study protocol was evaluated and approved by the Faculty of Medical Sciences of the National University of Asunción, by virtue of Resolution No. 0708 00 2022 of the Board of Directors of the Faculty of Medical Sciences of the National University of Asunción, article 2, which refers to the process ethical approval of non-experimental studies (Ethical Opinion Number:002_006_2023).

### Data analysis

Initially, descriptive analyses of the mean, standard deviation, skewness (As), and kurtosis (Ku) were estimated to assess the normality of the items, where values were considered adequate when As <  ± 2 and Ku <  ± 7 (Finney & DiStefano, 2006). To assess the internal structure, Exploratory Graph Analysis (EGA) was performed, a technique that estimates the number of dimensions in multivariate data using undirected network models. The EGA was executed using the Gaussian Graph Model (GGM), which was estimated using the graphical least absolute shrinkage and selection operator (GLASSO), a regularization method on the (inverse) covariance matrix that reduces coefficients and shrinks to zero, resulting in a sparse network structure (Friedman et al., 2008). Additionally, the Walktrap algorithm was employed to determine the number of factors or communities (Pons & Latapy, 2005). The combination of GLASSO and the Walktrap algorithm has shown a high precision (Christensen et al., 2023).

Within the network depiction, each item is represented by a node with connections between circles symbolizing the edges. These edges indicate partial correlations between pairs of items considering all items within the network.

Centrality indices, such as closeness and betweenness, were not employed in the study because they have different assumptions that are not met in network analysis in psychology and may lead to interpretation problems (Bringmann et al., 2019). Furthermore, the strength was not evaluated because its value has been shown to be influenced by various factors (Hallquist et al., 2021). As part of the solution, network loadings were proposed, defined as the standardization of node strength divided among the dimensions identified by EGA. This allows them to remain uninfluenced by other values, thereby providing precise measurements. Network loadings represent the unique contribution of each node in shaping a coherent dimension within the network. The cut-off points for the network loadings were established based on the guidelines developed by the simulation study. Specifically, the values of small (0.15), moderate (0.25), and large (0.35) network loadings were considered (Christensen & Golino, 2021b).

Reliability was assessed using the bootstrap exploratory graphical analysis (bootEGA) approach, employing two values: structural consistency, defined as the proportion of times each dimension estimated through EGA had the same item composition in a set of bootstrapped samples. Item stability indicates how often items are replicated in their empirically derived dimensions and other dimensions. For these estimations, an approach with 1000 replications was employed to determine the structural consistency and stability of the items, with values above 0.75 considered acceptable (Christensen & Golino, 2021a).

A network approach was employed to assess the measure invariance based on sex. Initially, the configural invariance was estimated by conducting an EGA for each group separately (men and women) to visually identify whether the nodes were partitioned into identical communities for each group. Additionally, findings from bootEGA in the total sample were utilized to evaluate whether the elements were consistently organized into the same communities and whether the number and structure of communities fluctuated (Jamison et al., 2022). Subsequently, metric invariance first estimates a network and computes the network loadings using the assigned community memberships from configural invariance. The difference between the assigned loadings of the groups was then calculated as an empirical value. Next, group memberships are permuted and networks are estimated iteratively based on these permutations. Subsequently, network loadings were computed, and the difference in loadings between permuted groups was calculated to create a null distribution. Empirical differences were compared to the null distribution using a two-tailed p-value to assess significance. Both uncorrected and false discovery rate-corrected p-values are provided, with uncorrected p-values flagged for significance along with the direction of the group differences. To determine metric invariance, the item values should be not significant (*p* > 0.05, adjusted *p* > 0.10) (Jamison et al., 2022). Scalar or strict invariance was not calculated because network models do not estimate latent variables; therefore, item means or residuals that are used to achieve scalar or strict invariance are not obtained for comparison.

Statistical analyses were performed using the packages "lavaan", "EGAnet", and "qgraph". R software (R Core Team, 2019) and the R Studio Team environment (2021) were used in all cases.

## Results

### Descriptive analysis

Table 2 shows that the average score of the scale items varies between 0.84 and 0.95. Regarding skewness and kurtosis values, it was observed that all items had adequate values (skewness <  ± 2; kurtosis <  ± 7). This indicates a univariate normal distribution of items (Finney & DiStefano, 2006).

**Table 2:** Item’s descriptive analysis

Items	*M*	*SD*	*g1*	*g2*
1	0.95	0.97	0.75	-0.48
2	0.88	0.98	0.84	-0.39
3	0.89	1.02	0.84	-0.53
4	0.84	0.99	0.91	-0.33

*M* Mean, *SD* Standard deviation, *g1* Asymmetry, *g2* Kurtosis

### Validity based on internal structure

**Fig. 1:**
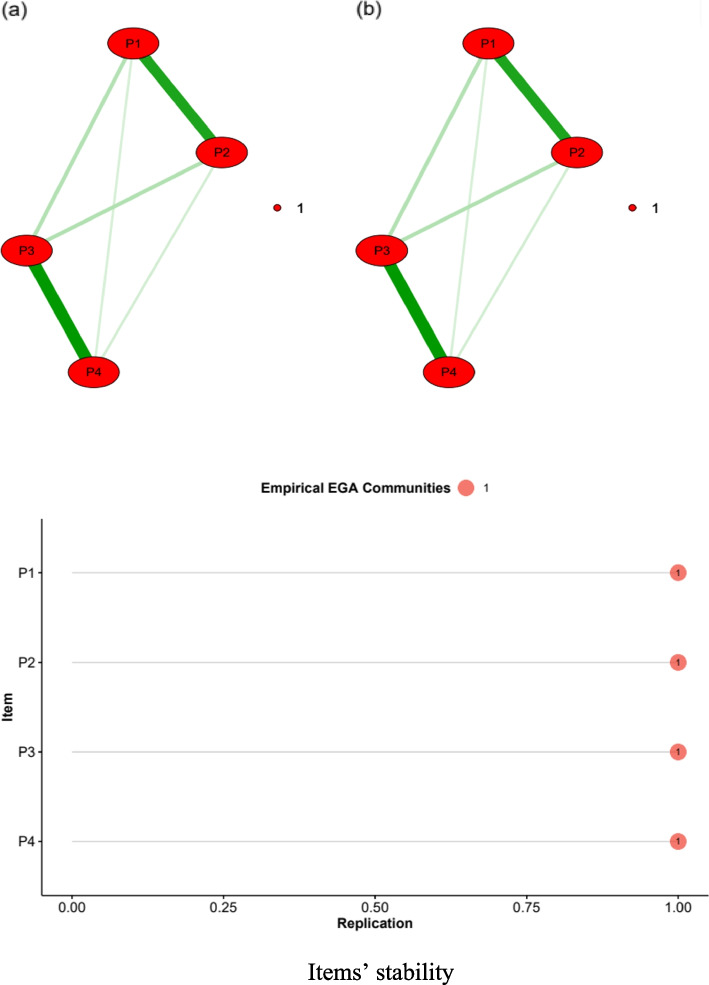
Dimensionality and stability of the PHQ-4 items Dimensionality using EGA (**a**) and bootEGA (**b**) of PHQ-4 items. P1 = nervousness (anxiety), P2 = worries (anxiety), P3 = loss of interest (depression), P4 = depressive mood (depression). Red edges represent positive relationships. Item = nodes, Replication = Proportion of times an item was assigned to the empirical EGA community in the bootstrap samples

Figure 1a shows the dimensionality estimated by the EGA, revealing a unidimensional structure comprising four nodes. These results were replicated 1000 times using a bootstrap that provided a unidimensional structure similar to that of the initial model (Fig. 1b). In addition, the network loading values were high for the items (P1 = 0.44, P2 = 0.45, P3 = 0.53, P4 = 0.44) (> 0.35). These values indicate the high contribution of each item to the development of a coherent dimension in the network.

### Reliability

Figure 1 shows that the stability of the items exceeded the acceptable threshold (≥ 0.75) and maintained their positions within the initial structure derived from the EGA. This examination highlighted the consistency of these items in their assignment to a unidimensional structure. Additionally, the observed structural consistency underscores the replication of the unidimensional design across all the examined instances, maintaining a perfect consistency rate of 100%. This emphasizes the high stability and coherence of node organization within the network across diverse iterations and sampling scenarios.

### Measurement invariance

**Fig. 2:**
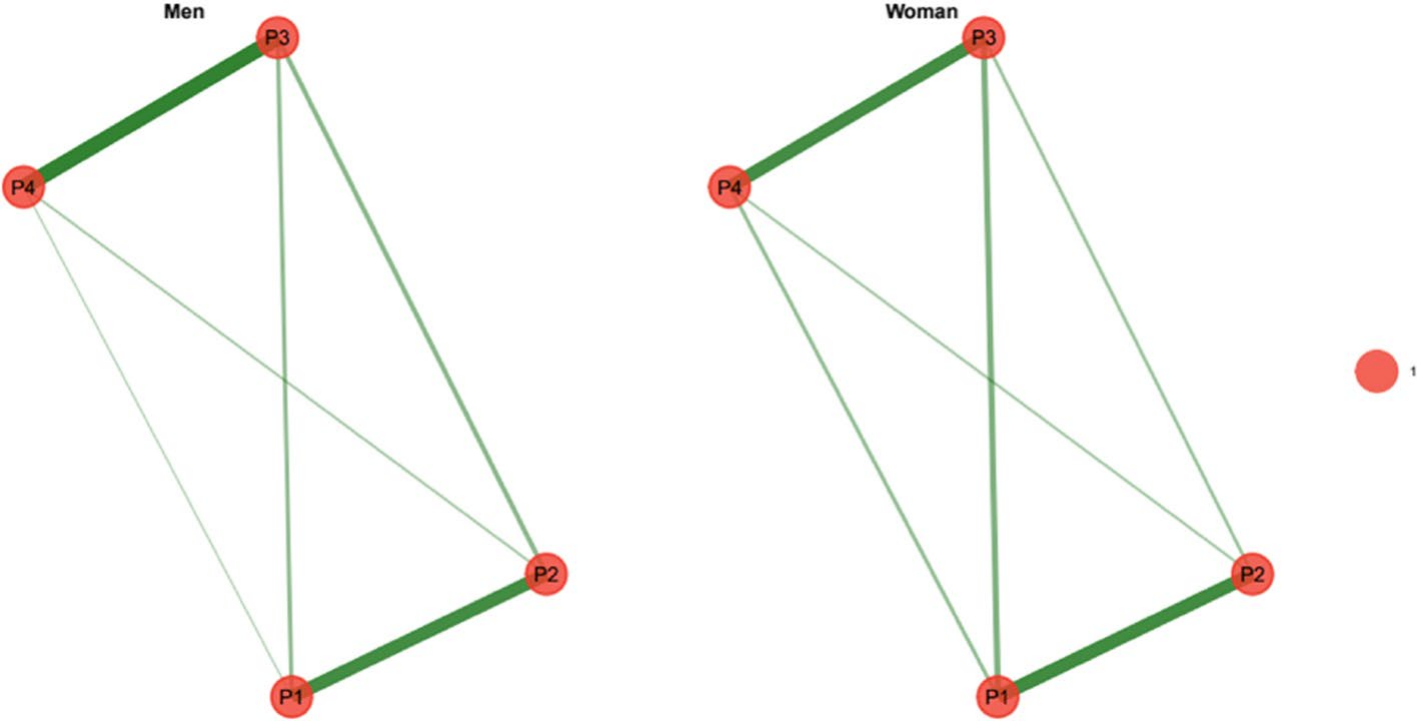
Configural invariance between men and women. EGA in men (left) and women (right)

In Fig. 2, the visual examination of EGA solutions for both men and women revealed comparable node partitioning into communities. Moreover, bootEGA conducted on the combined sample corroborated this finding, demonstrating a consistent clustering of nodes within the same communities across iterations (see Fig. 1). These results provide evidence for configural invariance. Subsequently, metric invariance analysis for EGA was conducted using permutation tests with sex as the grouping variable. Table 3 shows that the items had no significant differences (*p* > 0.05, adjusted *p* > 0.10) in network loading, indicating that the EGA structure functions similarly as a function of gender.

**Table 3:** Metric invariance according to sex

Items	Difference	*p*	p.adj	Significance
P1	-0.051	0.162	0.648	n.s
P2	0.033	0.386	0.772	n.s
P3	0.011	0.798	0.84	n.s
P4	0.01	0.84	0.84	n.s

*p p*-values, *p.adj p*-values corrected, *n.s.* not significant

## Discussion

To the best of our knowledge, the PHQ-4 does not present psychometric evidence in the Paraguayan context despite the significant percentage of people with severe symptoms of depression and anxiety (Caycho-Rodríguez et al., 2021; Torales et al., 2022a, 2022b). This necessitates the validation of measures of depression and anxiety. Therefore, this study aimed to evaluate the psychometric evidence of the Spanish version of the PHQ-4 in the Paraguayan sample factor using the psychometric NA approach.

Our literature review indicates that this is the first study to analyze the structure of PHQ-4 using a psychometric network approach. The results supported the one-factor structure using EGA with an adequate level of accuracy, based on high levels of stability. EGA has proven to be a useful procedure with many advantages over other exploratory methods, such as parallel analysis and exploratory factor analysis (Christensen, 2020). In addition, all network loadings were robust (Christensen & Golino, 2021a, 2021b). These results suggest that PHQ-4 items are facets of anxiety and depression that maintain a reciprocal relationship (Borsboom, 2017; Christensen et al., 2020). Thus, the concepts of anxiety and depression can be understood as a set of dynamic and interacting symptoms that form a system. That is, the connections between the nodes of the network would indicate that one item of the PHQ-4 influences and is influenced by the response levels of the other items.

The results provide a new analytical framework to conceptualize and interpret the PHQ-4 in the context of a Latin American country such as Paraguay. Initially, the proposed unidimensional structure of the PHQ-4 seemed divergent from the original model and supported by different studies, and was composed of two factors: depression and anxiety (Christodoulaki et al., 2022; Kazlauskas et al., 2023; Khubchandani et al., 2016; Kim et al., 2021; Kocalevent et al., 2014; Kroenke et al., 2009; Lenz & Li, 2022; Lopez Guerra et al., 2022; Löwe et al., 2010; Mendoza et al., 2022; Mills et al., 2015). However, since its construction, PHQ-4 assumes that symptoms of depression and anxiety coexist (Kroenke et al., 2009). This would demonstrate convergence with the initial theoretical assumption of the PHQ-4 and would make the unidimensional model a complementary version of the broader or general measurement of anxiety and depressive symptoms. Differences in the network structure of the PHQ-4 obtained in this study and those reported in previous studies may be attributed to the cultural characteristics associated with how populations experience anxiety and depression.

Reliability was assessed using the structural consistency method, which verified that all the data were organized within a single dimension from a set of replications. The single factor replicated accurately in 100% of the bootstrap samples and 100% of the derived dimensions. This indicates that the presence of a single factor is homogeneous even in the presence of other network structures. Previous studies estimated the reliability of the PHQ-4 using classical test theory and internal consistency measures such as Cronbach's alpha; therefore, the comparison of reliability estimation is limited. From network analysis, internal consistency measures do not allow us to determine whether items remain unidimensional within multidimensional models, whereas the structural consistency method indicates whether scales are unidimensional and internally consistent (Christensen et al., 2020).

When the unidimensional network structure was tested, evidence of configural invariance was reported, and the items did not show significant differences between men and women; therefore, the PHQ-4 structure works in the same way in both groups. In this sense, the network structure evaluates male and female groups in a similar way, which produces comparable results (van Borkulo et al., 2015). The presence of PHQ-4 measurement invariance between male and female groups has also been observed in previous studies using classical test theory methods both at the Latin American level (Kocalevent et al., 2014) and in other cultural contexts (Christodoulaki et al., 2022; Kazlauskas et al., 2023; Mendoza et al., 2022). In contrast, the assessment of network invariance included all network symptoms and relationships. This finding provides further support for the validity of the PHQ-4 as a self-reported screening measure, as it indicates that the network structure of anxiety and depression symptoms is comparable between the sexes. Therefore, possible differences in the frequency of anxiety and depression symptoms between men and women would express true symptom differences and would not be the product of a measurement method bias. Combining all the above findings that support the evidence of validity, reliability, and invariance of the Spanish version of the PHQ-4, the questionnaire appears to have adequate psychometric properties through network analysis.

The study had strengths, such as the large number of participants, the number of questions in the PHQ-4, and the use of novel statistical techniques. It has been suggested that the EGA procedure works adequately with sample sizes of 500 and above, with 90% accuracy (Golino & Epskamp, 2017). However, the results should be interpreted with consideration of a set of limitations. First, the participants were part of a convenience sample and therefore were not representative of the Paraguayan population. This generated a biased sample, where the majority of the participants were male, single, with completed university studies, permanent jobs, and living in an urban area of the city. This limits the generalizability of the sample findings to the population. It is advisable to test the network structure of anxiety and depression symptoms in more homogeneous groups of people. Second, we exclusively used self-reported measures to obtain data on depression and anxiety. This could lead to responses being influenced by common method biases such as social desirability (Podsakoff et al., 2003). Third, the data were limited to a cross-sectional design; therefore, the analyses were limited to the group level and to a single moment in time. Fourth, information was obtained only from those with access to the internet. However, many people in Paraguay do not have access to the Internet, and there is a significant gap between urban and rural areas (Villamayor, 2022). Fifth, the study only explored the equivalence of the network structure of anxiety and depression measures as a function of gender but did not do so between different age groups or other relevant characteristics, such as educational level. Assessing this equivalence could be important if researchers wish to use the PHQ-4 to compare anxiety and depression across these groups.

### Implicancias

These findings have theoretical and practical implications. First, the findings are useful for increasing the conceptual framework of anxiety and depression in the Paraguayan context, as they have implications for the identification of the relationships between depressive and anxiety symptoms, which are associated with the improvement or deterioration of mental health in Paraguay (Torales et al., 2021, 2022a, 2022b). The results also suggest that this assessment can cover specific symptoms or the general aspects of anxiety and depression. This is because PHQ-4 assumes the coexistence of depression and anxiety symptoms (Kroenke et al., 2009). Likewise, it should not be assumed a priori that psychometric measures such as the PHQ-4 present measurement equivalence between different groups. Therefore, the findings allow us to obtain information on the equivalence of the network structure between groups of men and women. Thus, the differences in the groups' symptom networks are attributable to the relationships between anxiety and depression symptoms, and are not a product of variations in the measurement instrument (Hirschfeld & Von Brachel, 2019). Thus, this equivalence would indicate that both groups understand the relationships between symptoms in the same way, which allows for comparative studies between both groups and corroborates the evidence provided by previous studies (Kaiser et al., 2021), despite the presence of biological and environmental differences associated with depression and anxiety. Furthermore, the use of psychometric networks does not force researchers to decode complex factor-loading matrices or identify the most appropriate type of rotation for the factor structure. This would help significantly reduce the biases and errors in the analyses (Golino et al., 2020).

On a practical level, a brief measure such as the PHQ-4 allows rapid measurement of the relationship between anxiety and depressive symptoms. In addition, the brevity of PHQ-4 allows its inclusion in more complex network models involving a large number of variables. However, psychometric networks facilitate the visual translation of information through network plots. This can be intuitively interpreted by health professionals and researchers with little experience in psychometric analyses. Finally, researchers and health professionals interested in identifying specific relationships or evaluating the efficacy of an intervention targeting specific anxiety and depression symptom relationships would find the PHQ-4 useful.

## Conclusion

The PHQ-4 presented optimal evidence of validity based on its internal structure, reliability, and invariance between sexes based on a psychometric network analysis. Therefore, PHQ-4 can be used as an accurate and brief measure of anxiety and depressive symptoms.

## Availability of data amd materials

The data presented in this study are available on request from the corresponding author.

## Abbreviations

PHQ-4: Patient Health Questionnaire-4
EGA: Exploratory Graph Analysis
GGM: A Gaussian Graph Model
GLASSO: Graphical least absolute shrinkage and selection operator
bootEGA: Bootstrap exploratory graphical analysis ()

## References

[CR1] Ahmadi SM, Arani AM, Bakhtiari M, Emamy MHD (2019). Psychometric properties of Persian version of patient health questionnaires-4 (PHQ-4) in coronary heart disease patients. Iranian Journal of Psychiatry and Behavioral Sciences.

[CR2] Arroll B, Goodyear-Smith F, Crengle S, Gunn J, Kerse N, Fishman T, Hatcher S (2010). Validation of PHQ-2 and PHQ-9 to screen for major depression in the primary care population. The Annals of Family Medicine.

[CR3] Bringmann LF, Elmer T, Epskamp S, Krause RW, Schoch D, Wichers M, Wigman JTW, Snippe E (2019). What Do Centrality Measures Measure in Psychological Networks?. Journal of Abnormal Psychology.

[CR4] Bock GR, Goode JA, Webb K (2003). The nature of intelligence.

[CR5] Bollen KA (2002). Latent variables in psychology and the social sciences. Annual Review of Psychology.

[CR6] van Borkulo C, Boschloo L, Borsboom D, Penninx BW, Waldorp LJ, Schoevers RA (2015). Association of symptom network structure with the course of depression. JAMA Psychiatry.

[CR7] Borsboom D (2008). Psychometric perspectives on diagnostic systems. Journal of Clinical Psychology.

[CR8] Borsboom D (2017). A network theory of mental disorders. World Psychiatry.

[CR9] Borsboom D, Cramer AO (2013). Network analysis: An integrative approach to the structure of psychopathology. Annual Review of Clinical Psychology.

[CR10] Borsboom D, Cramer AOJ, Kievit RA, Scholten AZ, Franić S, Lissitz RW (2009). The end of construct validity. The concept of validity: Revisions, new directions, and applications.

[CR11] Borsboom D, Mellenbergh GJ, van Heerden J (2003). The theoretical status of latent variables. Psychological Review.

[CR12] Canavire-Bacarreza, G., Recalde-Ramírez, L. (2022). *Salud mental en Paraguay: lo que revelan los datos*. Banco Mundial Blogs. https://blogs.worldbank.org/es/latinamerica/salud-mental-en-paraguay-lo-que-revelan-los-datos

[CR13] Caro-Fuentes, S., Sanabria-Mazo, J. P. (2023). A systematic review of the psychometric properties of the Patient Health Questionnaire-4 (PHQ-4) in clinical and non-clinical populations. Journal of the Academy of Consultation-Liaison Psychiatry. Available online. 10.1016/j.jaclp.2023.11.685. Accessed 27 Nov 2023 10.1016/j.jaclp.2023.11.685 38012988

[CR14] Cano-Vindel, A., Muñoz-Navarro, R., Medrano, L. A., Ruiz-Rodríguez, P., González- Blanch, C., Gómez-Castillo, M. D., Capafons-Bonet, A., Chacón, F., Santolaya, F., y PsicAP Research Group. (2018). A computerized version of the Patient Health Questionnaire-4 as an ultra-brief screening tool to detect emotional disorders in primary care. *Journal of Affective Disorders*, *234*, 247–255. 10.1016/j.jad.2018.01.030 10.1016/j.jad.2018.01.030 29549826

[CR15] Caycho-Rodríguez T, Tomás JM, Vilca LW, Carbajal-León C, Cervigni M, Gallegos M, Videla CB (2021). Socio-demographic variables, fear of COVID-19, anxiety, and depression: Prevalence, relationships and explanatory model in the general population of seven Latin American countries. Frontiers in Psychology.

[CR16] Christensen A (2020). Towards a network psychometrics approach to assessment: Simulations for redundancy, dimensionality, and loadings.

[CR17] Christensen AP, Garrido LE, Guerra-Peña K, Golino H (2023). Comparing community detection algorithms in psychometric networks: A Monte Carlo simulation. Behavior Research Methods.

[CR18] Christensen AP, Golino H (2021). Estimating the stability of psychological dimensions via bootstrap exploratory graph analysis: A Monte Carlo simulation and tutorial. Psych.

[CR19] Christensen AP, Golino H (2021). On the equivalency of factor and network loadings. Behavior Research Methods.

[CR20] Christensen AP, Golino H, Silvia PJ (2020). A psychometric network perspective on the validity and validation of personality trait questionnaires. European Journal of Personality.

[CR21] Christodoulaki A, Baralou V, Konstantakopoulos G, Touloumi G (2022). Validation of the Patient Health Questionnaire-4 (PHQ-4) to screen for depression and anxiety in the Greek general population. Journal of Psychosomatic Research.

[CR22] Constantin MA, Schuurman NK, Vermunt JK (2023). A general Monte Carlo method for sample size analysis in the context of network models.

[CR23] Costantini G, Perugini M, Rauthmann JF, Sherman R, Funder DC (2017). Network analysis for psychological situations. *The Oxford handbook of psychological situations*.

[CR24] Cramer AO, Van der Sluis S, Noordhof A, Wichers M, Geschwind N, Aggen SH, Borsboom D (2012). Dimensions of normal personality as networks in search of equilibrium: You can't like parties if you don't like people. European Journal of Personality.

[CR25] Dias SF, Gomes AA, Espie CA, Ruivo Marques D (2023). Analysis of the Psychometric Properties of the Glasgow Sleep Effort Scale Through Classical Test Theory, Item Response Theory, and Network Analysis. Sleep and Vigilance.

[CR26] Dominguez-Lara S, Merino-Soto C (2016). Sobre o uso do Little Jiffy na validação dos testes: Comentários a Ávila e colaboradores. Jornal Brasileiro De Psiquiatria.

[CR27] Epskamp S, Borsboom D, Fried EI (2018). Estimating psychological networks and their accuracy: A tutorial paper. Behavior Research Methods.

[CR28] Epskamp S, Fried EI (2018). A tutorial on regularized partial correlation networks. Psychological Methods.

[CR29] Epskamp S, Maris G, Waldorp LJ, Borsboom D, Irwing P, Booth T, Hughes DJ (2016). Network psychometrics. Wiley handbook of psychometric testing.

[CR30] Epskamp S, Rhemtulla M, Borsboom D (2017). Generalized network psychometrics: Combining network and latent variable models. Psychometrika.

[CR31] Ferguson C, Initiative ADN (2021). A network psychometric approach to neurocognition in early Alzheimer's disease. Cortex.

[CR32] Ferrando PJ, Anguiano-Carrasco C (2010). El análisis factorial como técnica de investigación en psicología. Papeles Del Psicólogo.

[CR33] Fonseca-Pedrero, E. (2018). Análisis de redes en psicología [Network analysis in psychology]. *Papeles del Psicólogo*, *39*(41), 1–12. 10.23923/pap.psicol2018.2852

[CR34] Finney SJ, DiStefano C, Hancock GR, Mueller RO (2006). Nonnormal and categorical data in structural equation modeling. Structural equation modeling: A second course.

[CR35] Friedman J, Hastie T, Tibshirani R (2008). Sparse inverse covariance estimation with the graphical lasso. Biostatistics.

[CR36] Ghaheri A, Omani-Samani R, Sepidarkish M, Hosseini M, Maroufizadeh S (2020). The four-item patient health questionnaire for anxiety and depression: A validation study in infertile patients. International Journal of Fertility & Sterility.

[CR37] Gilbody S, Sheldon T, House A (2008). Screening and case-finding instruments for depression: A meta-analysis. Canadian Medical Association Journal.

[CR38] Giuntoli L, Vidotto G (2021). Exploring Diener’s multidimensional conceptualization of well-being through network psychometrics. Psychological Reports.

[CR39] Golino HF, Demetriou A (2017). Estimating the dimensionality of intelligence like data using Exploratory Graph Analysis. Intelligence.

[CR40] Golino HF, Epskamp S (2017). Exploratory graph analysis: A new approach for estimating the number of dimensions in psychological research. PLoS ONE.

[CR41] Golino H, Shi D, Christensen AP, Garrido LE, Nieto MD, Sadana R, Thiyagarajan JA, Martinez-Molina A (2020). Investigating the performance of exploratory graph analysis and traditional techniques to identify the number of latent factors: A simulation and tutorial. Psychological Methods.

[CR42] Hajek A, König HH (2020). Prevalence and correlates of individuals screening positive for depression and anxiety on the phq-4 in the German general population: Findings from the nationally representative German socio-economic panel (GSOEP). International Journal of Environmental Research and Public Health.

[CR43] Hallquist MN, Wright AGC, Molenaar PCM (2021). Problems with Centrality Measures in Psychopathology Symptom Networks: Why Network Psychometrics Cannot Escape Psychometric Theory. Multivariate Behavioral Research.

[CR44] Hamilton M (1959). The assessment of anxiety states by rating. British Journal of Medical Psychology.

[CR45] Hamilton M (1960). A rating scale for depression. Journal of Neurology, Neurosurgery, and Psychiatry.

[CR46] Hartung TJ, Friedrich M, Johansen C, Wittchen HU, Faller H, Koch U, Mehnert A (2017). The Hospital Anxiety and Depression Scale (HADS) and the 9-item Patient Health Questionnaire (PHQ-9) as screening instruments for depression in patients with cancer. Cancer.

[CR47] Hevey D (2018). Network analysis: A brief overview and tutorial. Health Psychology and Behavioral Medicine.

[CR48] Hirschfeld G, Von Brachel R (2019). Improving Multiple-Group confirmatory factor analysis in R-A tutorial in measurement invariance with continuous and ordinal indicators. Practical Assessment, Research, and Evaluation.

[CR49] Jamison, L., Golino, H., Christensen, A. P. (2022). Metric Invariance in Exploratory Graph Analysis via Permutation Testing. *PsycArxiv*. 10.31234/osf.io/j4rx9

[CR50] Kaiser T, Herzog P, Voderholzer U, Brakemeier EL (2021). Unraveling the comorbidity of depression and anxiety in a large inpatient sample: Network analysis to examine bridge symptoms. Depression and Anxiety.

[CR51] Kazlauskas E, Gelezelyte O, Kvedaraite M, Ajdukovic D, Johannesson KB, Böttche M, Lotzin A (2023). Psychometric properties of the Patient Health Questionnaire-4 (PHQ-4) in 9230 adults across seven European countries: Findings from the ESTSS ADJUST study. Journal of Affective Disorders.

[CR52] Keith TZ, Caemmerer JM, Reynolds MR (2016). Comparison of methods for factor extraction for cognitive test-like data: Which overfactor, which underfactor?. Intelligence.

[CR53] Khubchandani J, Brey R, Kotecki J, Kleinfelder J, Anderson J (2016). The psychometric properties of PHQ-4 depression and anxiety screening scale among college students. Archives of Psychiatric Nursing.

[CR54] Kim HW, Shin C, Lee SH, Han C (2021). Standardization of the Korean version of the Patient Health Questionnaire-4 (PHQ-4). Clinical Psychopharmacology and Neuroscience.

[CR55] Kliem S, Moessle T, Klatt T, Fleischer S, Kudlacek D, Kroeger C, Wiltink J (2016). Psychometric evaluation of an Arabic version of the PHQ-4 based on a representative survey of Syrian refugees. Psychotherapie, Psychosomatik, Medizinische Psychologie.

[CR56] Kocalevent RD, Finck C, Jimenez-Leal W, Sautier L, Hinz A (2014). Standardization of the Colombian version of the PHQ-4 in the general population. BMC Psychiatry.

[CR57] Kroenke K, Spitzer RL (2002). The PHQ-9: A new depression diagnostic and severity measure. Psychiatric Annals.

[CR58] Kroenke K, Spitzer RL, Williams JB (2001). The PHQ-9: Validity of a brief depression severity measure. Journal of General Internal Medicine.

[CR59] Kroenke K, Spitzer RL, Williams JB, Löwe B (2009). An ultra-brief screening scale for anxiety and depression: The PHQ–4. Psychosomatics.

[CR60] Lenz AS, Li C (2022). Evidence for measurement invariance and psychometric reliability for scores on the PHQ-4 from a rural and predominately hispanic community. Measurement and Evaluation in Counseling and Development.

[CR61] Lloret-Segura S, Ferreres-Traver A, Hernández-Baeza A, Tomás-Marco I (2014). El análisis factorial exploratorio de los ítems: Una guía práctica, revisada y actualizada. Anales De Psicología.

[CR62] López Guerra V, Aguirre Mejia ÁJ, Guerrero Alcedo JM (2022). Propiedades psicométricas y estructura factorial del cuestionario de salud del paciente PHQ-4 en estudiantes universitarios ecuatorianos. Revista Cubana de Enfermería.

[CR63] Lovibond PF, Lovibond SH (1995). Manual for the Depression Anxiety Stress Scales.

[CR64] Löwe B, Wahl I, Rose M, Spitzer C, Glaesmer H, Wingenfeld K, Brähler E (2010). A 4-item measure of depression and anxiety: Validation and standardization of the Patient Health Questionnaire-4 (PHQ-4) in the general population. Journal of Affective Disorders.

[CR65] Luke DA, Harris JK (2007). Network analysis in public health: History, methods, and applications. Annual Review of Public Health.

[CR66] Mahmud S, Mohsin M, Dewan MN, Muyeed A (2023). The global prevalence of depression, anxiety, stress, and insomnia among general population during COVID-19 pandemic: A systematic review and meta-analysis. Trends in Psychology.

[CR67] Materu J, Kuringe E, Nyato D, Galishi A, Mwanamsangu A, Katebalila M, Wambura M (2020). The psychometric properties of PHQ-4 anxiety and depression screening scale among out of school adolescent girls and young women in Tanzania: A cross-sectional study. BMC Psychiatry.

[CR68] McFarland D (2020). The effects of using partial or uncorrected correlation matrices when comparing network and latent variable models. Journal of Intelligence.

[CR69] McNally RJ (2016). Can network analysis transform psychopathology?. Behaviour Research and Therapy.

[CR70] Mendoza NB, Frondozo CE, Dizon JIWT, Buenconsejo JU (2022). The factor structure and measurement invariance of the PHQ-4 and the prevalence of depression and anxiety in a Southeast Asian context amid the COVID-19 pandemic. Current Psychology.

[CR71] Mills SD, Fox RS, Pan TM, Malcarne VL, Roesch SC, Sadler GR (2015). Psychometric evaluation of the patient health questionnaire–4 in Hispanic Americans. Hispanic Journal of Behavioral Sciences.

[CR72] Mitchell AJ (2010). Short screening tools for cancer-related distress: A review and diagnostic validity meta-analysis. Journal of the National Comprehensive Cancer Network.

[CR73] Mitchell AJ, Coyne JC (2007). Do ultra-short screening instruments accurately detect depression in primary care? A pooled analysis and meta-analysis of 22 studies. British Journal of General Practice.

[CR74] Mitchell AJ, Vaze A, Rao S (2009). Clinical diagnosis of depression in primary care: A meta-analysis. The Lancet.

[CR75] Mulvaney-Day N, Marshall T, Downey Piscopo K, Korsen N, Lynch S, Karnell LH, Ghose SS (2018). Screening for behavioral health conditions in primary care settings: A systematic review of the literature. Journal of General Internal Medicine.

[CR76] Ohayon MM, Hong SC (2006). Prevalence of major depressive disorder in the general population of South Korea. Journal of Psychiatric Research.

[CR77] Parmentier H, Garcia-Campayo J, Prieto R (2013). Comprehensive review of generalized anxiety disorder in primary care in Europe. Current Medical Research and Opinion.

[CR78] Podsakoff PM, MacKenzie SB, Lee J-Y, Podsakoff NP (2003). Common method biases in behavioral research: A critical review of the literature and recommended remedies. Journal of Applied Psychology.

[CR79] Pons P, Latapy M (2005). Computing communities in large networks using random walks. Lecture Notes in Computer Science. Lecture Notes in Computer Science.

[CR80] Pons P, Latapy M (2006). Computing communities in large networks using random walks. Journal of Graph Algorithms and Applications.

[CR81] R Core Team (2019). A language and environment for statistical computing.

[CR82] R Studio Team (2021). A language and environment for statistical computing.

[CR83] Renovanz M, Soebianto S, Tsakmaklis H, Keric N, Nadji-Ohl M, Beutel M, Hickmann AK (2019). Evaluation of the psychological burden during the early disease trajectory in patients with intracranial tumors by the ultra-brief Patient Health Questionnaire for Depression and Anxiety (PHQ-4). Supportive Care in Cancer.

[CR84] Schmank CJ, Goring SA, Kovacs K, Conway AR (2019). Psychometric network analysis of the Hungarian WAIS. Journal of Intelligence.

[CR85] Schmittmann VD, Cramer AO, Waldorp LJ, Epskamp S, Kievit RA, Borsboom D (2013). Deconstructing the construct: A network perspective on psychological phenomena. New Ideas in Psychology.

[CR86] Schumann I, Schneider A, Kantert C, Löwe B, Linde K (2012). Physicians’ attitudes, diagnostic process and barriers regarding depression diagnosis in primary care: A systematic review of qualitative studies. Family Practice.

[CR87] Serrano-Blanco A, Palao DJ, Luciano JV, Pinto-Meza A, Luján L, Fernández A, Haro JM (2010). Prevalence of mental disorders in primary care: Results from the diagnosis and treatment of mental disorders in primary care study (DASMAP). Social Psychiatry and Psychiatric Epidemiology.

[CR88] Soares GH, Santiago PHR, Werneck RI, Michel-Crosato E, Jamieson L (2021). A psychometric network analysis of OHIP-14 across Australian and Brazilian populations. JDR Clinical & Translational Research.

[CR89] Spitzer RL, Kroenke K, Williams JB, Löwe B (2006). A brief measure for assessing generalized anxiety disorder: The GAD-7. Archives of Internal Medicine.

[CR90] Thompson C, Kinmonth AL, Stevens L, Pevele RC, Stevens A, Ostler KJ, Campbell MJ (2000). Effects of a clinical-practice guideline and practice-based education on detection and outcome of depression in primary care: Hampshire Depression Project randomised controlled trial. The Lancet.

[CR91] Torales J, Barrios I, Ayala N, O’Higgins M, Palacios JM, Ríos-González C, Ventriglio A (2021). Ansiedad y depresión en relación a noticias sobre COVID-19: un estudio en población general paraguaya. Revista de salud pública del Paraguay.

[CR92] Torales J, Barrios I, O'Higgins M, Almirón-Santacruz J, Gonzalez-Urbieta I, García O, Ventriglio A (2022). COVID-19 infodemic and depressive symptoms: The impact of the exposure to news about COVID-19 on the general Paraguayan population. Journal of Affective Disorders.

[CR93] Torales J, Torres-Romero AD, Di Giuseppe MF, Rolón-Méndez ER, Martínez-López PL, Heinichen-Mansfeld KV, Ventriglio A (2022). Technostress, anxiety, and depression among university students: A report from Paraguay. International Journal of Social Psychiatry.

[CR94] Ventura-León, J., Sánchez-Villena, A. R., Caycho-Rodríguez, T. (2023). Validity Evidence and Reliability of a Subjective Well-Being Scale: A Psychometric Network Analysis. *Trends in Psychology*, 1–15. 10.1007/s43076-022-00251-x 10.1007/s43076-022-00251-x PMC9810381 40479407

[CR95] Villamayor LE (2022). Brecha digital y el acceso a internet en el Paraguay como derecho fundamental en tiempos de COVID-19. Ciencia Latina Revista Científica Multidisciplinar.

[CR96] Wicke FS, Krakau L, Löwe B, Beutel ME, Brähler E (2022). Update of the standardization of the Patient Health Questionnaire-4 (PHQ-4) in the general population. Journal of Affective Disorders.

[CR97] Wittchen HU, Pittrow D (2002). Prevalence, recognition and management of depression in primary care in Germany: The Depression 2000 study. Human Psychopharmacology: Clinical and Experimental.

[CR98] World Health Organization. (2017). World Mental Health Day 2017 - Mental health in the workplace. https://www.who.int/news-room/events/detail/2017/10/10/default-calendar/world-mental-health-day-2017

[CR99] Zung WW (1965). A self-rating depression scale. Archives of General Psychiatry.

[CR100] Zung WW (1971). A rating instrument for anxiety disorders. Psychosomatics.

